# Older adults’ long-term engagement in self-managed fall prevention exercise: a qualitative longitudinal study of the digital Safe Step intervention

**DOI:** 10.1186/s12877-025-06776-x

**Published:** 2025-11-28

**Authors:** Beatrice Pettersson, Sara Lundell, Åsa Audulv, Lillemor Lundin-Olsson, Marlene Sandlund

**Affiliations:** 1 Department of Community Medicine and Rehabilitation, Umeå University, Umeå, Sweden; 2 Department of Sociology and Department of Community Medicine and Rehabilitation, Umeå University, Umeå, Sweden; 3 Department of Nursing, Umeå University, Umeå, Sweden

**Keywords:** Aged, Exercise, Behavior change, Fall prevention, E-health, Self-management, Longitudinal study, Qualitative research

## Abstract

**Background:**

Falls among community-dwelling older adults can be significantly reduced through exercises for balance and strength. Digital solutions show promise in increasing the reach and promote adherence to fall prevention exercises among older adults. However, research on long-term engagement in self-managed fall prevention programs is lacking. The Safe Step application is designed, in collaboration with older adults, to motivate and support them in independently engaging in balance and strength exercises. The aim of this study was to explore longitudinal patterns of older adults’ engagement in self-managed fall prevention exercise supported by the Safe Step digital application.

**Methods:**

A qualitative longitudinal study was nested within a randomized controlled trial that evaluated the effectiveness of the Safe Step application in reducing falls among community-living older adults. A selection of participants who maintained an exercise dose of ≥ 60 min each week was invited to the study. Fifteen participants were included. Each participant was interviewed twice, first at the end of a twelve-month trial period and then after an additional six months. The analysis followed the Pattern-Oriented Longitudinal Analysis approach, analyzing patterns of change over time.

**Results:**

Four engagement patterns were identified that began to emerge during the first year and were consolidated over time: (i) Coherent and sustained pattern, (ii) Coherent and episodic pattern, (iii) Integrated and sustained pattern, and (iv) Integrated and episodic pattern. The long-term engagement in self-managed digital fall prevention was characterized by the degree of cohesion and regularity in training. Initially, all participants followed the exercise recommendations provided by the application. With time they developed different strategies to maintain the exercises that resonated with their own preferences and daily activities.

**Conclusions:**

The digital program played a meaningful role in initiating and establishing exercise routines, while other determinants also influenced long-term engagement strategies. Support for self-management of fall preventive exercise needs to evolve over time to meet the changing needs of individuals and their different patterns of exercise engagement. Further research is needed to inform digital interventions aimed at supporting long-term engagement in fall prevention programs.

## Background

Falls among older adults are a global public health priority due to their high prevalence and severe consequences [1]. Annually, one-third of community living older adults (≥ 65 years) sustain a fall, and the risk increases with age. Falls can result in severe physical consequences, such as fractures, and can therefore impact both health and quality of life [2]. Falls can also cause a fear of falling, which can limit participation in daily activities and ultimately lead to a loss of independence and social isolation [3]. Due to the high prevalence of falls among older adults, these incidents also account for a significant proportion of older adults’ healthcare utilization and are one of the most common causes of mortality [4]. Thus, preventing falls among older adults is of major importance to both individuals and society.

As a result of substantial research, it has been established that performing balance and strength training can reduce the rate of falls among community-dwelling older adults. However, the effect varies depending on the type of exercises, intensity, and duration [5]. Global guidelines encourage adults over the age of 65 to engage in balance and functional strength exercises at least three times per week, progressively increasing in intensity over a minimum of 12 weeks and continuing longer for greater effects [6]. However, maintaining the recommended exercise dose over time has been shown to be challenging, regardless of whether the interventions have been delivered supervised by a health professional [7], individually, or in a group setting [8].

Barriers to adherence to fall prevention exercise have been addressed in several studies. Common barriers to participate in group exercise classes for fall prevention include transportation limitations, lack of access, time constraints, financial considerations, and an aversion to comparing one’s functional capacity with others [9]. Participation in supervised fall prevention exercise is also often time-limited, and transitioning from supported exercise to self-management has been shown to be challenging [10]. On the other hand, home-based fall prevention exercise can present barriers such as difficulties in remembering how to perform the exercises, experiencing a lack of feedback on performance, and a lack of social support [11]. Nevertheless, in a large survey of willingness to attend different fall prevention activities, home-based strength and balance training was by far the most favored activity [12]. The incorporation of balance and strength exercises into everyday situations at home has also been shown to improve adherence [13]. Home-based training presents a practical choice that can entail lower costs for both the individual and society. However, to have a substantial impact on public health, effective ways to reach out with and maintain fall prevention exercise must be presented.

The use of mobile health (mHealth) applications can vastly increase the reach of fall prevention exercise interventions. mHealth applications have the ability to provide continuous support for individuals to be able to initiate and adapt exercises according to their own capability and prerequisites. Several studies have explored the ability of digital technology to support older adults’ adoption and maintenance of exercise. Better results have been found for technology in which behavior change techniques and theoretical models have been incorporated into the mHealth applications [14, 15]. Results from qualitative studies highlight that visual guidance in exercise videos is considered especially supportive for feeling confident in exercise performance [14, 16, 17]. While mHealth applications may support older adults in adopting and maintaining fall prevention exercises in the short term, little is known regarding the factors that contribute to successful long-term engagement beyond the trial period [18].

The Safe Step application aims to provide accessible fall prevention exercises to many older adults. The application supports the adoption of balance and strength exercises through educational videos in home environments and facilitates integration into daily routines with integrated behavior change support [19]. Our previous studies have determined that the application is perceived as supportive of developing personalized exercise routines and confidence in performing them [11] but also supports motivational determinants important for behavior change [14]. A qualitative study with participants who had exercised with the Safe Step application for one year showed that the Safe Step application acted as an important external mediator to support development of fall prevention exercise habits [20]. When addressing the research gap regarding older adults’ long-term engagement in fall prevention exercise and especially when digital technology is used, it is crucial to also explore their engagement on a long-term basis. Therefore, the aim of this study was to explore longitudinal patterns of older adults’ engagement in self-managed fall prevention exercise supported by the Safe Step digital application.

## Methods

### Study design

This qualitative longitudinal research (QLR) study was nested within a randomized controlled trial (RCT) [21]. Data was collected via repeated individual interviews. The first interview was conducted immediately upon completion of the 12-month trial period and the second was conducted after an additional six months. QLR is useful for exploring experiences and health behaviors across time, and, for example, understanding facilitators and inhibitors in transitions [22]. In this study, we use the Pattern-Oriented Longitudinal Analysis approach (POLA) which is one way of analyzing qualitative longitudinal data materials [23]. The POLA approach was developed to focus on variation in change in a data material, as opposed to identifying one uniform changing process. Thus, more complexities and contextual factors can be accounted for, for example various ways of changing behavior can be described [23]. The study was reported in accordance with reporting guidelines for qualitative research [24] and qualitative longitudinal research [25].

### Study context

The participants had been enrolled in the intervention group of the Safe Step RCT [21]. In the RCT, recruitment, registration, and data collection were digitized and managed via the project website and e-mail. The inclusion criteria for participating in the RCT were: being 70 years or older; having fallen or experienced a decline in perceived postural balance during the previous year; having access to a smartphone or tablet and using it regularly; having and using a personal email address; being able to understand verbal and written instructions in Swedish; being able to rise from a standard height chair without a person helping; and being able to walk independently indoors without a walking aid. The exclusion criteria were: having a progressive disease likely to cause a decline in strength or balance over the next year; experiencing memory dysfunction that affects everyday life activities; or taking part in more than three hours per week of strenuous high intensity exercise. The intervention group received the Safe Step application (v2), and monthly educational videos about healthy ageing and fall prevention. They exercised with the Safe Step application for twelve months. At the start of the trial, the participants composed their own individual exercise program in the Safe Step application supported by a brief introduction video. They were instructed to choose one exercise that was sufficiently challenging from each of the ten categories of balance and strength exercises. The participants were advised to exercise for at least 30 min, three times per week, and to progress their program if they found the initially selected exercises becoming less challenging. The application also offered suggestions on how to integrate the exercises into everyday activities and how to perform them outdoors. Additionally, the application provided support for self-management of the exercise such as exercise planning and positive feedback after registering exercise [19].

### Recruitment and sample

Recruitment for the interview study was performed during the last six months of the data collection of the RCT. We applied a purposive sampling strategy, with the inclusion criterion of a reported exercise of ≥ 60 min per week with the Safe Step application at the 12-month follow-up. No exclusion criteria were applied. All participants who met the inclusion criteria (*n* = 45) were invited by email to participate in individual interviews via telephone or conference call. One reminder was sent. Nineteen participants accepted the invitation, one participant declined (reason unknown), and 25 participants did not respond. Of the 19 participants who participated in the first interview, 15 could be reached and were interviewed a second time. Thus, 15 participants (represented by 30 interviews) were included in this QLR study.

### Data collection

All individual interviews [26] were conducted by the author BP, a postdoctoral researcher who had previous experience with qualitative interviewing. She had no previous relationship with the participants, and the participants were only informed that she was employed within the study. The first set of interviews was conducted between September 2021 and March 2022 and lasted 27–91 min (median 64 min). The second set of interviews was conducted between March 2022 and September 2022 and lasted 16–87 min (median 35 min). Most of the interviews were conducted by telephone (*n* = 27), with a few by video conference call (*n* = 3) based on participants’ individual preferences.

Semistructured interview guides with open-ended questions were developed and discussed by the authors. The first interview guide included questions about previous exercise experience, motivation for exercise, exercise with the Safe Step application, perceived effects of the exercise on health and quality of life, and digital competency. Findings from these interviews have also been included in a study focusing on the development of exercise habits, and the full interview guide is available in that publication [20]. For the second round of interviews, the questions focused primarily on the period that had passed since the first interview to capture changes over time while addressing the same content areas. Before these interviews were conducted, the first author (BP) carefully reviewed each participant’s first interview transcript to ensure continuity and to avoid unnecessary repetition. This made it possible to adapt the interviews to the individual participants, for example by following up on aspects from the previous interview (e.g., “During the last interview, you told me that you used the app to plan your exercise and that the reminders were good support. Do you still use the app?”), as suggested by Kneck and Audulv [23]. All interviews were audio recorded and transcribed verbatim by a professional transcriber.

### Data analysis

The analysis was conducted according to the POLA approach as described by Kneck and Audulv [23], and qualitative content analysis [27] was used during the first phase of the analysis. The POLA approach is appropriate for analyzing various patterns of change over time, and the findings typically present a number of different ways the participants changed over time [23]. Thus, has the POLA approach similarities with between and within case analysis [28], however the focus is not on the case itself but on how cases change over time. The analysis was conducted using the software MAXQDA 2022.

**Fig. 1:**
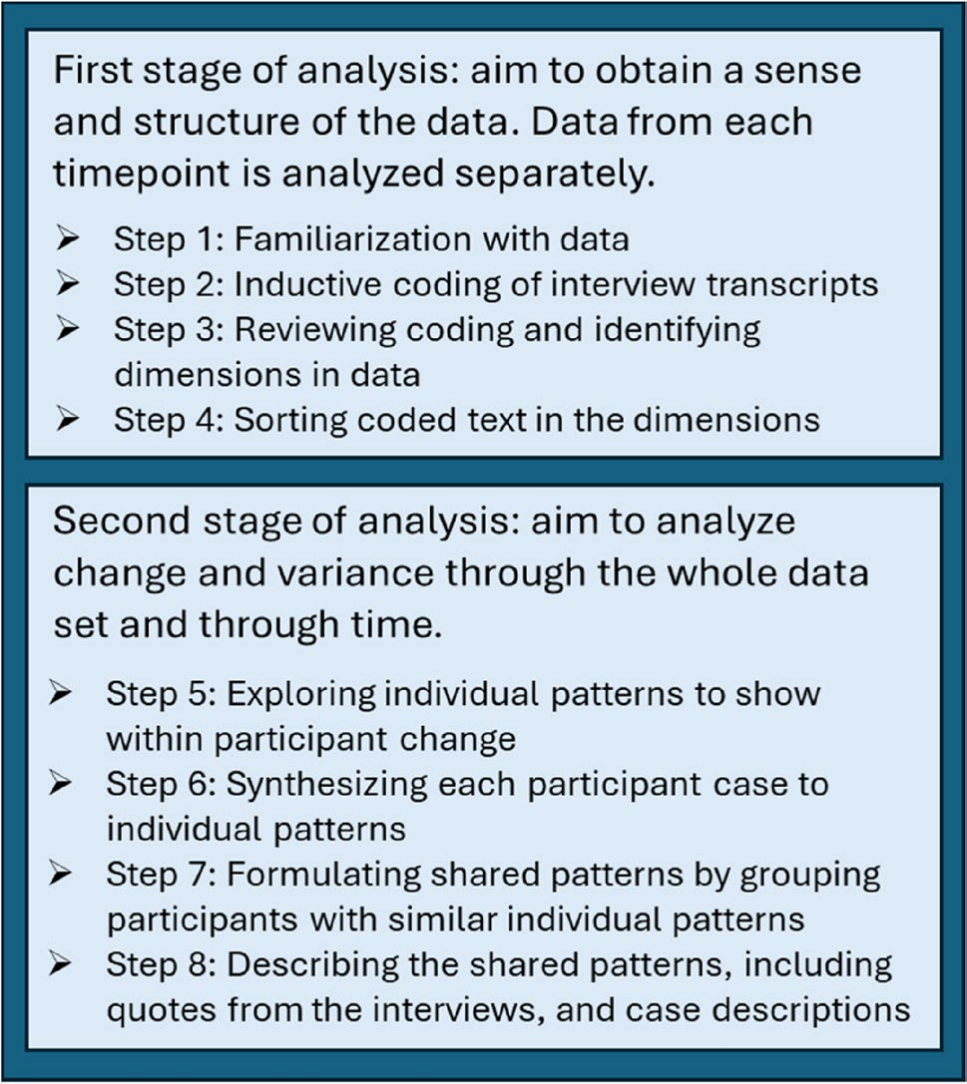
Schematic description of the analysis process

SL led the data analysis in close collaboration with BP and ÅA, who were involved in all stages of the analysis process (i.e., coding, constructing matrices and regular discussions about the evolving analysis). The data were analyzed inductively in a diachronic manner, i.e., all the data were collected before the data was analyzed [22]. The transcripts from both the first and the second interviews for all fifteen participants were included in the analysis. As recommended by Kneck and Audulv [23], the analysis was performed in a two-stage process (Fig. 1). The first stage was conducted to gain an initial understanding and preliminary structure of the data. The first step consisted of familiarization, i.e., reading the transcripts several times (SL). In the next step, the transcripts were inductively coded (SL, BP, ÅA) according to qualitative content analysis [27]. In the third step, the coding was reviewed and discussed among all the authors, and three preliminary dimensions were identified in the data: (1) *Driving forces*,* thoughts*,* and insights*; (2) *The person’s described exercise activities*; (3) *Experienced* s*upport from the app.* In step four, all text in the interview transcripts were sorted into these three dimensions (SL).

In the second stage, the longitudinal aspect, i.e., changes over time and variance in the data was the focus. This was done by visualizing and interpreting data using matrices [23]. In step five, the changes in individual participants were in focus, and one matrix was constructed for each participant (SL, ÅA, BP), and were used to visualize change and/or lack thereof between the participants’ first and second interviews. The matrices included condensed interview text for each of the three dimensions, sorted by first and second interview. In step six, a summary for each domain was synthesized to illustrate individual patterns, i.e., the participants’ paths through time (SL). In step seven, the whole research team conducted a workshop, in which the participants were analytically grouped on the basis of the characteristics of their individual patterns. By clustering participants with similar paths through time, four shared patterns were identified. In step eight, SL and BP prepared a preliminary description of the characteristics of each shared pattern, which was then discussed within the research team, and examined in relation to the data. This process led to some revisions of the shared patterns before the final results were formulated. The final results included case descriptions to exemplify change over time for participants within each shared pattern.

The research team consisted of four physiotherapists and one nurse, with collective expertise in older adults’ health, fall prevention, eHealth, and self-management of physical activity. All had extensive experience in interview studies and qualitative methodologies, and two had prior experience with QLR studies. All the researchers were women, three had an insider perspective (e.g., were involved in the development and evaluation of Safe Step), and two had an outsider perspective.

## Results

The interviewed participants had a mean age of 75.8 years and were predominantly women (87%). They were accustomed to technology and had various experiences of physical activity and exercise prior to the study (Table 1). The characteristics of those who did not respond to the follow-up interview invitation did not clearly differ from those who accepted in terms of baseline characteristics or habits of using the application during the trial.

**Table 1:** Self-reported baseline participant characteristics from the RCT, categorized by the patterns in this QLR study

Interviewee	Gender	Age (Years)	Health in general	Walking aid	Use of internet on smartphone or tablet	Number of falls previous year	Physical activity (min/week)	Strenuous physical activity (min/week)	TTM
**Coherent and sustained pattern**									
ID 1	W	82	Very good	N	Almost every day	0	> 120	0	Maintenance
ID 3	W	82	Good	Y	Multiple times per day	2	> 120	0	Maintenance
ID 6	W	81	Good	N	Multiple times per day	0	30–59	1–29	Precontemplation
ID 15	W	70	Poor	Y	Almost every day	1	1–29	0	Precontemplation
ID 18	W	71	Fair	N	Almost every day	0	30–59	0	Contemplation
**Coherent and episodic pattern**									
ID 7	W	87	Good	N	Almost every day	1	> 120	0	Maintenance
ID 9	W	80	Very good	N	Multiple times per day	1	> 120	0	Maintenance
ID 13	W	76	Fair	N	At least once per week	1	30–59	0	Precontemplation
ID 17	W	71	Good	Y	Almost every day	2	0	0	Contemplation
**Integrated and sustained pattern**									
ID 2	W	73	Good	N	Multiple times per day	3	60–89	60–89	Contemplation
ID 14	W	74	Fair	N	Multiple times per day	2	30–59	1–29	Contemplation
ID 16	M	70	Fair	N	Multiple times per day	3	30–59	1–29	Preparation
ID 19	W	77	Good	N	Multiple times per day	0	> 120	30–59	Action
I**ntegrated and episodic pattern**									
ID 8	M	72	Fair	Y	Almost every day	3	60–89	90–120	Maintenance
ID 10	W	71	Good	N	Almost every day	5	> 120	60–89	Maintenance

*W *Woman, *M* Man, *Y* Yes, *N* No, *TTM* Trans Theoretical Model*

*Precontemplation = Not engaging in regular exercise and no intention to start in the future, Contemplation = Not exercising yet but committed to take action within six months, Preparation = Seriously considering starting exercising – has taken some steps toward the objective, Action = Exercising consistently but for less than six months, Maintenance = Exercising consistently for six months or more

The analysis showed that the participants shared the experience of having impaired balance and acknowledged the importance of exercise in improving balance. They described how they initially followed the recommendations in the Safe Step application, i.e., they performed a program of ten exercises three times a week and adjusted their exercises when they were found to be less enjoyable or not strenuous enough. After some favorite exercises were found, they mainly continued using these exercises for the remainder of the period. Four exercise patterns were identified. These describe how engagement in self-managed digital fall prevention exercise was changed during the one-year trial period and six months thereafter. The patterns started to emerge during the first year and were consolidated over time. These patterns were characterized by the degree of cohesion and regularity in training, although some characteristics were common to all exercise patterns. The patterns were: (i) *Coherent and sustained pattern; (ii) Coherent and episodic pattern;* (iii) *Integrated and sustained pattern; and (iv) Integrated and episodic pattern*. The findings section is organized to give an in-depth presentation of each pattern, illustrated with quotes and a case description. The characteristics of each pattern are also summarized in Table 2, which provides an overview and a comparison of the patterns.

**Table 2:** Characteristics of the four patterns of engagement in self-managed digital fall prevention exercise

	Sustained	Episodic
**Coherent**	• Performed a program of their selected exercises in one session to get through the exercise promptly. • Performed their exercise program regularly several times a week.	• Performed a program of their selected exercises in one session to get through the exercise promptly. • Performed their exercise program sporadically and/or with periods of exercise breaks.
**Integrated**	• Integrated exercises into everyday activities, i.e., performed a few chosen exercises triggered by other activities, environment, or body sensations. • Performed their exercises regularly, almost every day.	• Integrated exercises into everyday activities, i.e., performed a few chosen exercises triggered by other routines, environment, or body sensations. • Performed their exercises sporadically and the number each week varied.

### Coherent and sustained pattern

Five participants employed a coherent and sustained pattern of engagement. At the end of the trial, all of them were set on maintaining their exercise routines. These participants continued with a selected program of exercises to perform three times a week or daily. Some participants consistently used the same exercises, whereas others introduced changes every so often, choosing from their own exercise battery. Despite maintaining a coherent exercise pattern over time, a few week-long breaks were described. To establish a clear exercise routine, participants in this pattern often linked their exercise to an already established daily routine, such as performing the full program immediately after dressing in the morning or scheduling it for specific weekdays and times. This helped them to carry out all the exercises on one occasion and maintain consistency over time.

The Safe Step application was considered valuable support to be able to maintain this pattern and was said to compensate for personal shortcomings. For instance, those who struggled with establishing exercise routines experienced the reminders supportive. For others, exercise registration and the subsequent positive feedback were important to maintain motivation. Another perspective was that during periods of feeling lonely, especially during the COVID-19 pandemic, the older adults demonstrating the exercises in the app were regarded as companies.

*"The reminders are definitely my greatest support feature of the app. I think they are essential for maintaining my exercise routine. Without them, I believe I would relapse again. [They are] truly very important. Crucial, I would say."*

*[ID 1, interview 2]*

The perceived benefits of the application’s support motivated the participants to continue to use the application after the trial ended. Despite the crucial support provided by the reminders, some participants still discontinued using them after the trial ended.

During and after the trial period, most participants in this pattern combined the Safe Step exercises with other physical activities, such as walking, other home training, or exercise classes. They varied in terms of previous experiences of physical activity and exercise. Some saw it as a routine, whereas others viewed it as a necessary inconvenience and needed strong incentives to initiate and complete their exercise. The participants described how they were able to adapt their exercise routines to life’s demands, especially those with a long history of physical activity. A shared experience was that exercising with the Safe Step application had contributed to improved balance and strength, increased confidence in their balance, and even regained vitality.

*"Here, you get a bit of joy from life too, you know, by moving. The body needs to move, you know."*

*[ID 15, interview 2]*

An important driving force for the participants to join the study and maintain their exercise routines was their desire to remain independent. Moreover, participating in the study could contribute to a feeling of being seen, and that someone cared for and showed an interest in them as individuals.

**Table:** 

Textbox 1. Case description of a person with a coherent and sustained pattern of engagement	
Anna	
At the start of the study, Anna chose ten exercises in the Safe Step app. She has kept the same exercises for the entire 18 months since she wants to know them by heart. Every Monday, Wednesday, and Friday, she receives a reminder from the app and performs the exercises. Describing herself as lazy, she finds the reminders essential to maintaining her engagement over time. During the trial period, she registered her exercise in the app to get the following praise, which helped motivate her. By the end of the trial, she stopped registering the exercise but kept the reminders. She fears her exercise routine would falter without them. Previously, Anna attended a local gym but found the training boring and struggled to continue. Instead, she started taking walks, but she admits that these could be more frequent. Anna joined the Safe Step study since she thought it would help her with her balance issues. She says that physical activity does not come naturally to her, and she needs incentives to exercise. During the study, she experienced improvement in her balance and strength, leading to a greater sense of security. The improvements motivate her to continue her exercise routine.	

### Coherent and episodic pattern

Four participants followed a coherent and episodic pattern of engagement, either completing the entire program or selecting a few exercises. During the trial, some participants initially experimented with integrating the exercises into daily activities but ultimately settled into the coherent engagement pattern. The episodic approach was often due to competing demands from other exercises or daily tasks.

*”Yeah*,* but there was a period when I didn’t do anything at all*,* you know… instead*,* I did more gardening work. And then when I came inside*,* I was tired*,* and then I didn’t feel like doing anything more*,* just relaxing.”*

[ID 13, interview 2]

Participants who stopped exercising when the trial ended started again, for example, when experiencing a decline in physical function or when contacted for the second interview. The participants in this pattern emphasized the significance of their commitment to the study for maintaining their exercise regimen, citing a sense of obligation as a key motivator for adhering to the exercise recommendations.

*”I might feel guilty if I don’t do it… Towards the study. … I mean*,* when you know someone is following up*,* it feels… you’re more inclined to do things. It’s like when you… if you get some recommendations from a physiotherapist or something like that*,* then you train better when you know you have to demonstrate in a week or two.” *

[ID 13, interview 1]

During the trial, the participants found the Safe Step app crucial for completing their exercises, providing essential structure and support. While several app features, such as reminders and positive feedback, were valued or even a prerequisite during the trial, some participants discontinued their use of the app after the trial ended. Some continued watching exercise videos as they appreciated the clear instructions for performance.

Participants in this pattern had various experiences with physical activity, ranging from lifelong engagement to feeling compelled to train. Initially, the participants expressed anticipating improvements from the exercise. However, when improvements did not occur as expected, they prioritized the maintenance of physical capacity instead.

**Table:** 

Textbox 2. Case description of a person with a coherent and episodic pattern of engagement	
Elsa	
Elsa decided to join the Safe Step study to compensate for a group training that was halted due to the pandemic. She started performing the Safe Step exercises three times a week, but rather quickly reduced it to ‘a few days a week’. Initially, she changed her exercises but eventually settled into the same exercises. After the trial ended, she took a break from her balance training. However, after a few weeks she started to experience a decline in strength and decided to start the training again. This time she has selected a few exercises that she performs now and then while watching her favorite program on TV. Throughout the trial, Elsa relied on the app’s reminders to stay on track with her exercise. However, once the trial concluded, she chose to disable the notifications to avoid the feeling of constant alerts. Despite knowing her exercises by heart, she still enjoys watching the exercise videos while performing them. Elsa prioritizes enjoyment to stay motivated in her exercise routine. However, she finds the Safe Step exercises at home monotonous and challenging. Despite occasional attempts to alleviate her guilt towards the researchers, she has begun substituting these exercises with activities like walking and gardening. Initially, hopeful for balance improvement, Elsa has now adjusted her expectations to ‘at least not become worse’.	

### Integrated and sustained pattern

Four participants employed an integrated and sustained pattern of exercise engagement. Early in the trial, they shifted from completing the entire program in one session to incorporating exercises into daily activities. They selected favorite exercises that they found fun and challenging, which were triggered by other activities, environments, or bodily sensations.

"*So, I can sit in a chair and then get up and feel… yeah, there’s a bit of creaking in the thigh muscles and stuff like that. Well okay then, let’s do a session. I’ll do sit-to-stand from the chair, and while I’m sitting anyway, I’ll do some heel and toe movements and those kinds of things […] So that it’s sort of incorporated into everyday life.*" 

[ID 14, interview 2]

At the end of the trial period, the participants considered it important to continue exercising but revised their routines without the study’s obligations. They became more flexible with exercise days and focused on favorite exercises. Typically, they performed a few exercises daily, varying them throughout the week. The exercises were described as becoming as natural a routine as other activities.

*“It’s kind of the same thing*,* it’s not always fun to brush your teeth at night*,* same with this*,* it’s just part of everyday life*,* you know. So*,* there’s not much to think about*,* like should I*,* shouldn’t I*,* it’s just a given.”*

[ID 2, interview 1]

The participants in the integrated and sustained pattern emphasized the importance of continuing fall prevention exercises long-term. One strategy ivolved memorizing the exercises to reduce the need for conscious thought during execution, making it easier to maintain the exercise regimen. The Safe Step app was seen as helpful for integrating these exercises into daily life during the trial, although its usage varied. Most app features were used primarily during the trial and were no longer needed afterward.

The participants described increased awareness of fall prevention and promoted it within their social circles. They combined Safe Step exercises with other activities, mainly outdoors, e.g., walking in the woods or going to an outdoor gym. Participants with an integrated and sustained pattern expressed always having been physically active, valuing it for their well-being and health. They experienced improvements in strength and balance attributed to the Safe Step exercises, particularly during the trial, which motivated continued participation. The exercises were seen as crucial for fall prevention and enhancing leisure activities, such as confidently picking mushrooms in the woods. Despite severe health issues such as falls or strokes, participants adapted and continued their exercises to suit their new conditions.

**Table:** 

Textbox 3. Case description of a person with an integrated and sustained pattern of engagement	
Clara	
Initially, Clara performed the exercises coherently three times a week. During the trial, she gradually spaced them out and integrated them into daily activities. After the trial, she continued doing what she considers the most useful exercises, further integrating them into her routine, such as doing toe raises while watching TV or taking extra laps on the stairs. By the second interview, she practiced balance exercises daily, triggered by situations or bodily sensations, like stiffness. She appreciated the Safe Step app for its reminders and variety of exercises and kept it for exercise inspiration. Clara described an active lifestyle, including walking, biking, and outdoor gym workouts, emphasizing that physical activity had always been crucial to her. She believed exercise was essential for maintaining function and independence with age. She noted balance improvements during the trial and experienced that she had maintained them over time, although she still faced balance issues. The risk of falling motivated her to continue the exercises. Clara described that the trial heightened her awareness of how to prevent falls.	

### Integrated and episodic pattern

Two participants followed an integrated and episodic pattern, performing a few exercises they found useful very sporadically. Their decision to exercise was triggered by factors like a suitable song, decreased strength, different environments (e.g., stairs or beach), or routines (e.g., dog walks or brushing teeth). They experienced little or no balance improvement and gradually switched to other physical activities.

*“I was most diligent in the beginning*,* and then I kind of lost it a bit when I stopped noticing any more improvements.”*

The participants in this pattern did not consider the application crucial for maintaining their exercise. During the trial, they memorized or wrote down their routines on paper instead of using the app. While the reminders were found to be partially helpful, no one used them after the trial. They found registering their exercises and receiving feedback tiresome and impersonal. The exercise repository was either found to be a useful reminder or had little value.

During the COVID-19 pandemic, the Safe Step exercise was considered an important alternative to other forms of exercise. The participants described how they appreciated doing some exercises and physical activity outdoors in general, e.g., in the garden or in the woods, benefiting from natural balance training. At first, they saw potential in the Safe Step exercises, noticing small improvements. However, when progress plateaued their motivation declined. Nevertheless, they concluded that the exercise was important for maintaining physical function and preventing decline.

*“But nothing is progressing. I think I would have deteriorated more quickly if I hadn’t done anything. I don’t know. […] I don’t find it particularly enjoyable*,* and I don’t feel like I see any changes from it.”*

[ID 8, interview 2]

One of the participants in the pattern experienced exercise as boring but important, whereas the other had extensive experience in various exercise forms but stressed that exercise should be enjoyable. However, they agreed on the importance of exercise to maintain their physical health, and health issues affecting their physical ability motivated them to do some exercise, even if it was boring.

**Table:** 

Textbox 4. Case description of a person with an integrated and episodic pattern of engagement	
Monica	
Monica describes herself as clumsy, and she has fallen a few times during the last year, but without injuring herself. Previously, she has tried other fall prevention exercise programs. She decided to try exercising with Safe Step because it was a research project, and she wanted new inspiration and input for balance training. Hower, she had rather low expectations and found exercising alone at home with the Safe Step exercises boring compared to exercise classes. During the trial, she exercised regularly with Safe Step, to ‘not let the researchers down’ and found the app useful for learning the exercises. Monica used the notifications and registered her exercises but stopped using the app when the trial ended. At that time, she planned to continue with the exercises more integrated in everyday life but was vague on how. By the follow up, she had resumed group exercises and took daily walks, occasionally performing some Safe Step exercises during her walks. She also did other balance exercises, both independently and in her group exercise class. She never really felt that her balance improved during the trial and thinks that an objective balance test would have been motivating.	

### Comparison of patterns

As shown above, the four patterns had different characteristics, with participants experiencing different facilitators and challenges in fall prevention exercise. At baseline, the participant characteristics were largely similar across the four patterns (Table 1). However, differences included a greater fall risk the year before the trial in both the integrated groups and greater engagement in strenuous physical activity before the study compared with the coherent groups. Previous experiences of physical activity varied among the four patterns. Even though the support from the Safe Step application was perceived important, sometimes even crucial, many participants stopped using the app after the trial. The integrated patterns seem more associated with doing exercises outdoors. Experienced improvements in strength and balance were more common in the sustained patterns, while a lack of improvement challenged motivation and led to substituting the Safe Step exercises with other activities. Table 3 summarizes the similarities and differences between the patterns.

**Table 3:** Similarities and differences between the patterns during the 18 months of the study

	Coherent and sustained pattern	Coherent and episodic pattern	Integrated and sustained pattern	Integrated and episodic pattern
**Previous experience of physical activity**	Variation within the group. Ranging from considering oneself lazy to having a history of extensive physical activity.	Variation within the group. Ranging from considering oneself lazy to having an active lifestyle.	Consistency in the group. Always been physically active, physical activity important for wellbeing.	Variation within the group. Ranging from seeing exercise as boring but important to having a history of being active as long as exercise is fun.
**Support from the app during the trial period**	- Reminders used to establish exercise routines. - Exercise registration and feedback giving encouragement. - The exercise program used as a checklist. - Exercise films used as company.	- Reminders used as a prerequisite for maintaining the exercise. - Exercise registration and feedback creating appreciation of accomplishment.	- Exercise films giving valuable information during exercise. - Reminders used to be aware of time for exercise. - Registration gives a sense of accomplishment.	- Reminders partly used as support to get exercise done. - Exercise program partly used as checklist.
**Use of app after the trial period**	Yes, all used exercise films or reminders.	Some stopped using the app, while some used exercise films or reminders.	Some stopped using the app, while some use exercise films and registration.	No use of the app.
**Experienced improvements**	Improved strength and balance.	No clear improvements.	Improved strength and balance.	No clear improvements.
**Driving force for continued training**	Experienced improvements and continue being independent.	Commitment to the study and no decline physical function.	Experienced improvements, preventing falls and increasing participation in leisure activities.	Not decline in physical function.
**Safe Step vs. other activities**	Complementing Safe Step exercise with other physical activities.	Replacing some Safe Step exercise with other physical or daily activities.	Complementing Safe Step exercise with other physical activities.	Replacing some Safe Step exercise with other physical activities.
**Setting for exercise/physical activity**	Mostly indoors, combined with outdoors (i.e., walking).	Mostly indoors, combined with outdoors (i.e., walking and gardening).	Mainly outdoors.	Indoors and outdoors.

## Discussion

In this study, we aimed to explore patterns of adults’ long-term engagement in self-managed digital fall prevention exercise. The analysis suggested that ongoing engagement with the Safe Step application was characterized by the cohesion and consistency of exercise routines demonstrated in four different patterns. Each pattern employs varying strategies for initiating and maintaining the digitally supported fall prevention exercise. There is a shortage of evidence regarding the long-term effects of fall prevention exercise, maintenance of gained benefits, or changes in physical activity, health behaviors, and health status beyond the end of falls prevention exercise interventions [29], especially digital ones. Many qualitative studies with older adults’ experiences of digital fall prevention exercise address experiences related to the end of trials [30]. Therefore, this study contributed to our understanding of how longitudinal behavioral patterns of exercise engagement develop among older adults and the defining characteristics of these patterns.

A defining characteristic of the exercise patterns observed in our study was the underlying motivation that sustained participants’ engagement in their exercise routines over time. In the sustained exercise patterns, participants experienced physical improvements during the trial, which motivated them to continue exercising after the trial concluded. In the episodic patterns, improvements were not as evident, so expectations were adjusted, and they expressed the importance of at least maintaining physical function and health. Still, for both of these patterns, the exercise was valued and endorsed personal goals [31]. Another form of motivational regulation that played a significant role for the participants was when the exercise aligned with their personal identities and values [31]. The participants employing an integrated and sustained pattern, identified themselves as active people which made it easier to integrate balance exercises in their everyday life. Research indicates that motivational regulations where the activity is externally regulated but still holds high personal value can play a greater role in sustaining physical activity than intrinsic motivation does, especially when the physical activity and exercise may not be inherently enjoyable in the short term [32]. Moreover, according to Self-determination theory [33], individuals internalize or take ownership of an important but unenjoyable behavior when it has personal meaning and significance [34], such as expressions of perceived health benefits, improved physical function, and independence in our study. Previous research confirms these factors as important reasons for older adults to carry out fall prevention training, not solely for the purpose of preventing falls [35, 36]. Additionally, for older adults with coherent and episodic exercise patterns an additional external motivation was a commitment to research, reflecting a broader desire to contribute to understanding older adults’ health [33]. Similar results have repeatedly been shown to support short-term positive effects on motivation, but these effects are not sustained [37, 38]. This could therefore explain some of the participants’ discontinuation of using the Safe Step application after the trial had ended.

In our study, participants demonstrated strong intentions and awareness of the benefits of exercise. However, despite including the most active individuals, many still did not achieve the trial-recommended exercise duration of 90 min per week. Instead, the majority exercised for approximately 60 min per week, with only a few maintaining the previously established exercise regimen after the trial concluded. Furthermore, although participants completed the full program comprising 10 exercises, it progressively became shorter, averaging only 20 min instead of the intended 30 min. Adoption and retention in fall prevention exercise interventions for older adults are notoriously challenging [8], prompting numerous studies to explore barriers to adherence and design interventions supporting long-term maintenance. Current evidence advocates a minimum of three hours of balance and strength exercise per week to achieve the best fall prevention effect [5]. Consequently, a potential disparity exists between strategies that older individuals are willing to consider for fall prevention and evidence-based approaches. Older adults may lack motivation for regular strength and balance exercises solely for fall prevention, often preferring activities that align with personal goals, values, and interests [9]. Additionally, a lack of variability of exercises can also decrease motivation for long-term engagement with self-managed fall prevention exercise [11, 14]. From a clinical perspective, it becomes crucial to determine an effective but feasible amount of balance and strength exercise per week, considering that older adults should engage in various physical activities as part of a healthy lifestyle [1].

The participants expressed finding the app’s various features was helpful in maintaining their exercise routines during the trial. For instance, receiving notifications was seen as particularly helpful for exercise maintenance. In accordance with our findings, a systematic review by Jakob et al. [39] examined short-term adherence to various self-management apps and reported that push notifications and the ability to personalize the app were positively associated with adherence. Nevertheless, many participants in our study stopped using the app after the trial ended. Instead, some transitioned to other forms of exercise, whereas others integrated their learned exercises into daily activities, indicating that they had developed lasting exercise habits. This integration facilitated adherence to the exercises but also presented challenges in accurately assessing the time dedicated to balance and strength exercises. Indeed, there is a risk that excessive integration may lead to the partial maintenance of an exercise program or prescribed dose, potentially reducing its overall effectiveness. Considering these findings, it is essential to design digital programs that provide long-term support. With different approaches available, such as prescribed exercise templates (e.g., Standing Tall [40]) or more flexible routines (e.g., LiFE [41]), further studies are needed to determine which type of support is most effective from a long-term perspective and how it can be individualized depending on maintenance patterns.

On the basis of our results and recognizing the dynamic nature of self-management, adjustments to support may be necessary as individuals’ needs and preferences evolve. The advancement of techniques and theoretical frameworks in artificial intelligence (AI) allows for novel approaches to the automated tailoring of mHealth apps to an individual’s behavior and characteristics, thus potentially improving the effectiveness of the intervention. Alhussein and Hadjileontiadis [42] recommended the integration of AI and machine learning to personalize mHealth apps to support self-management. Just-In-Time Adaptive Intervention (JITAI) is an emerging technique with great potential to support health behavior by providing the right type and amount of support at the right time [43]. However, a systematic literature review has shown that JITAI interventions for physical activity and reducing sedentary behavior are in their early stages and are most commonly designed for people between 20 and 60 years of age [44]. Consequently, further research is needed to design self-managed fall prevention exercise interventions that can adapt to older adults’ individual patterns of engagement.

### Methodological considerations

This study has several strengths. To the best of our knowledge, this is the first study providing insight into long-term maintenance of digitally supported fall prevention exercise for older adults, with interviews conducted at the end of the one-year trial and then after an additional six months. During recruitment for the first interview, we intentionally included a larger number of participants than we expected to follow up, in order to account for potential dropouts before the second interview. As four individuals did not respond to the invitation to the second interview, 15 participants were finally included in the study. In line with the concept of information power as described by Malterud et al. [45], this sample was considered sufficient, as adequacy depends not only on sample size but also on the study aim, sample specificity, quality of dialogue, and analysis strategy. However, since one pattern included only two participants, we recognize that the study would have benefited from a few additional participants. Per the longitudinal design, we performed two individual interviews with each of the 15 participants, allowing us to obtain rich personal narratives [46, 47]. The POLA method [23] makes it possible to explore changes in behavioral patterns over time, deepening the understanding of participants’ long-term engagement.

Initially, the inclusion criterion was to invite participants who adhered to the RCT’s recommendation of 90 min of exercise per week. However, the inclusion criterion had to be changed, as few participants met the recommendation. Future research will determine whether these patterns also apply to participants with lower or higher exercise durations/frequencies.

The participants in this qualitative study were primarily women (87%). This reflects the gender distribution observed not only in the Safe Step randomized controlled trial [48] but also in fall prevention studies in general [29]. However, we had planned for a more balanced inclusion of both genders in the study and invited a proportionally greater number of men (10 out of 45). Nonetheless, the final distribution of participants ended up with predominantly women and it would have been valuable to have more perspectives from men on long-term engagement in self-managed and digital fall prevention exercise [9]. A strength of the study is that participants were recruited from across Sweden, providing a variation of experiences from different contexts. Another strength is the use of a qualitative longitudinal approach, which allowed us to capture changes in behavior and motivation over time and to provide nuanced and contextual insights into participants’ engagement, knowledge that could not be obtained from survey data alone. Moreover, the findings are closely tied to the Safe Step application. However, we believe that the results of this study are novel and can be of value to understand behavioral patterns after a fall prevention intervention, both in self-managed digital exercise programs and in more traditional supervised formats.

A strength of the data analysis process is that researcher triangulation was used in all phases of the analysis to obtain credibility, where all authors were involved in reading and coding transcripts and in regular discussions of the steps of the analysis process. According to the COREQ checklist [24], member checking is often recommended as a way to enhance credibility in qualitative research. In this study, however, no member checking was conducted. Routine application of member checking has been increasingly questioned in the methodological literature [49, 50]. Instead, several scholars argue that member checking is not relevant to all qualitative research and should only be used when it serves a clear purpose for the study design and research questions [49, 50]. In our case, the analysis process was conducted collaboratively within the research team, with continuous discussions and comparison between emerging analysis and interview transcripts. We therefore judged that additional member checking with participants was not necessary to strengthen the credibility of the findings,

The COVID-19 pandemic began during the recruitment period for the Safe Step RCT. We observed a higher level of recruited participants following the announcement of restrictions for adults over 70 years in Sweden [48]. Therefore, during the interviews conducted at the end of the trial, the impact of the pandemic on their exercise routines was explored. While some reported that exercising with Safe Step replaced other forms of training and made it easier to exercise during the pandemic, others did not believe that the pandemic influenced their decision to participate in the study or their exercise with the application at all [20]. Therefore, it cannot be ruled out that participants’ long-term experience of exercising with the Safe Step app was influenced by the societal factors resulting from the pandemic. The pandemic also highlighted that digital technology is a valuable tool for delivering health interventions to the population and is not restricted to in-person meetings.

## Conclusion

This study sheds light on the longitudinal engagement patterns observed among older adults utilizing a self-managed digital fall prevention application, the Safe Step application. The findings delineate four distinct patterns, characterized by varying levels of cohesiveness and consistency in long-term engagement with fall prevention training through the app. Our results demonstrate the meaningful role that technology can play in initiating and establishing routines, although it is not the sole determinant of long-term engagement strategies. Moreover, our findings underscore the need for exercise prescribers to be aware of different patterns of long-term exercise engagement, and that support for self-management of exercise may need to meet evolving needs of individuals over time. These insights emphasize the need for further research to refine our understanding and inform the development of effective interventions aimed at sustaining long-term engagement in self-managed digital fall prevention programs.

## Data Availability

The data generated and analyzed in the current study are not publicly available due to confidentiality towards the participants but are available from the corresponding author on reasonable request.

## Abbreviations

AI: Artificial Intelligence
JITAI: Just-In-Time Adaptive Intervention
mHealth: Mobile Health
POLA: Pattern-Oriented Longitudinal Analysis
QLR: Qualitative Longitudinal Research
RCT: Randomized Controlled Trial
